# Thrombo-inflammatory prognostic score can predict the outcome of stroke: a retrospective cohort study

**DOI:** 10.3389/fnagi.2024.1391559

**Published:** 2024-05-30

**Authors:** Xingyu Zhu, Lin Lan, Yi Liu, Na He, Jie Wu, Yingqiang Guo, Hong Li, Dongze Li

**Affiliations:** ^1^Department of Cardiovascular Surgery, West China School of Medicine, West China Hospital, Sichuan University, Chengdu, China; ^2^Cardiovascular Surgery Research Laboratory, West China Hospital, Sichuan University, Chengdu, China; ^3^Department of Emergency Medicine, West China School of Nursing, West China Hospital, Sichuan University, Chengdu, China; ^4^Nursing Key Laboratory of Sichuan Province, Chengdu, China; ^5^Department of Emergency Medicine, West China Tianfu Hospital, West China Hospital, Sichuan University, Chengdu, China

**Keywords:** thrombo-inflammatory prognostic score, TIPS, ischemic stroke, outcome, prognosis, emerging risk assessment tool

## Abstract

**Introduction:**

Inflammatory and thrombotic biomarkers are simple prognostic indicators of adverse clinical outcomes in patients with ischemic stroke (IS). However, isolated assessment of inflammatory or thrombus biomarkers in patients with IS is limited in clinical practice.

**Methods:**

This study aimed to evaluate the predictive value of a novel, simplified thrombo-inflammatory prognostic score (TIPS) that combines both inflammatory and thrombus biomarkers in the early phase of IS and to identify high-risk patients at the time of admission. The study population comprised 915 patients with a primary diagnosis of IS in the emergency departments of five grade A tertiary hospitals in China.

**Results:**

Patients were divided into two groups based on the modified Rankin Scale (mRS): <3 and ≥3. TIPS with a value of “2” indicates biomarkers for high inflammation and thrombosis, “1” represents a biomarker, and “0” signals the absence of a biomarker. Multivariate logistic regression analysis was employed to identify the association between TIPS and clinical outcomes. TIPS was an independent predictor of unfavorable functional outcomes and mortality. It had a superior predictive value for clinical outcomes compared to the National Institutes of Health Stroke Scale (NIHSS) (effect ratio, 37.5%), D-dimer (effect ratio, 12.5%), and neutrophil-to-lymphocyte ratio (effect ratio, 25%).

**Conclusion:**

The survival probability of TIPS with a score of 0 is twice as high as that of TIPS with a score of 2. The survival rate for TIPS with a score of 1 is one time higher than that for TIPS with a score of 2. The predictive value of TIPS for unfavorable functional outcomes is represented by an AUC of 0.653. TIPS is associated with an increased risk of death and unfavorable functional outcomes in patients with IS and may be a useful tool for identifying high-risk patients at the time of admission.

## 1 Introduction

Ischemic stroke (IS) is characterized by the sudden loss of blood flow to a specific area of the brain, resulting in impaired neurological function. Globally, one in six individuals experiences a stroke in their lifetime, with more than 13.7 million people suffering from stroke each year, leading to 5.8 million deaths annually (Herpich and Rincon, 2020). Findings from a prospective national hospital cohort study in China, examining stroke mortality, disability, and recurrence rates 12 months after the first stroke, indicated an in-hospital mortality rate of 0.9% [95% confidence interval (CI): 0.8%–1.1%] for IS. The 12-month mortality rate for discharged patients with IS was 6.0% (95% CI: 5.7%–6.3%) (Tu et al., 2021). During the early stages of hospital admission, conducting high-risk assessments, categorizing patients, and promptly implementing the corresponding treatment measures have significant potential to improve patient prognosis and conserve healthcare resources (Saini et al., 2021). Consequently, research has focused on early recognition and prevention.

In the prediction of IS risk, researchers commonly use various biomarker tools to assess patients’ risk levels (Kamtchum-Tatuene and Jickling, 2019). These tools include serum, imaging, inflammatory, coagulation, and metabolic biomarkers, among others. Each tool has its unique advantages and limitations, suitable for different types of patients and clinical scenarios (Planas, 2018). For example, serum biomarkers like C-reactive protein (CRP) and brain natriuretic peptide (BNP) have a high correlation with stroke risk. Inflammatory biomarkers like white blood cell count (WBC) and neutrophil-lymphocyte ratio (NLR) reflect inflammation levels (Siwicka-Gieroba et al., 2019). Coagulation biomarkers such as prothrombin time (PT) and activated partial thromboplastin time (APTT) reflect coagulation function and have a high correlation with stroke risk (Lisman, 2018). Metabolic biomarkers like blood glucose and cholesterol levels reflect metabolic status and have a high correlation with stroke risk (Kernan et al., 2014; Hou et al., 2021).

However, the current biomarkers and assessment tools fall short of their required clinical value. Our preliminary study showed that the combined use of thrombo-inflammatory markers can provide more prognostic information than a single thrombus or validated marker does (Li et al., 2023). The thrombo-inflammatory prognostic score (TIPS) stands out as an emerging risk assessment tool with unique advantages. TIPS combines inflammatory (e.g., WBC and CRP) and coagulation (e.g., PT and APTT) biomarkers to provide a comprehensive assessment of patients’ inflammatory and coagulation states, offering more comprehensive risk prediction information. Thus, combining multiple biomarkers as clinical parameters may provide greater predictive value in understanding IS risk than relying on a single inflammatory or thrombotic biomarker. In this multicenter retrospective cohort study of patients with IS from the Retrospective Multicenter Study for Ischemic Stroke Evaluation (REMISE) study, we tested the hypothesis that the thrombo-inflammatory predictive scoring system (TIPS) could effectively stratify patients with IS, thereby enhancing the understanding of IS risk at the time of admission.

## 2 Materials and methods

### 2.1 Study design

The REMISE study was a multicenter retrospective cohort study registered at www.chictr.org.cn (ID: ChiCTR2100052025). Patients with IS were recruited from the emergency departments of five grade A tertiary hospitals in Sichuan, China, from January 2020 to December 2020. The study was conducted in accordance with the principles of the Declaration of Helsinki. The research protocol was approved by the Human Ethics Committee of the West China Hospital of Sichuan University (Approval Number of the Ethics Committee: 2021–1175). This was a retrospective chart review that did not require informed consent.

### 2.2 Study population

We included patients who were first diagnosed with IS according to the 2019 American Heart Association Stroke Guidelines and had a time from symptom onset to hospitalization of less than 6 h. Our exclusion criteria included: (1) individuals with a diagnosis of subarachnoid hemorrhage or transient ischemic attack; (2) malignant tumors; (3) severe liver or kidney dysfunction; (4) history of clinical signs of infection on admission or 30 days before IS onset; and (5) unavailable data to obtain Protein-Nutrition Index, Controlling Nutritional Status, or Geriatric Nutritional Risk Index scores on admission.

### 2.3 Data collection and measures

Experienced physicians used standard case report forms to retrieve the demographic and clinical data of patients during hospitalization from the REMISE research database. The data collected from the electronic health records included patient age, sex, vital signs, laboratory examination results, body mass index, medical history, arterial blood gas analysis, imaging examination results, adverse outcomes, and treatment received during hospitalization and at discharge. All laboratory and imaging examinations were conducted in accordance with the standard procedures of West China Hospital of Sichuan University.

Stroke-related neurological deficits at the time of admission were assessed using the National Institutes of Health Stroke Scale (NIHSS) (Powers et al., 2019). The NIHSS score ranges from 0 to 42, with higher scores indicating more severe neurological impairment. The A^2^DS^2^ score (ranging from 0 to 10) was calculated based on age, dysphagia, male sex, atrial fibrillation, and stroke severity (Li et al., 2014). The A^2^DS^2^ score is a clinical scoring system used to assess the risk of early recurrent stroke in patients who have experienced acute IS. The Pneumonia Severity Index (PSI) is a scoring system used to evaluate the severity of pneumonia and is calculated based on the following factors: age, sex, nursing home, disease, physical examination results, and laboratory and imaging results (Fine et al., 1997).

### 2.4 Outcomes

The primary research outcome measure was mortality, and the secondary outcome was unfavorable functional outcome, which was calculated using the modified Rankin Scale (mRS) and PSI.

### 2.5 TIPS

The TIPS used in our study was based on biomarker measurements of inflammation [WBC count, procalcitonin (PCT), interleukin-6, CRP, neutrophil count, lymphocyte count, and NLR] and thrombosis [platelet (PLT), pulmonary embolism, international standardized ratio (INR), activation time of local thrombotic tissue (APTT), and fibrinogen] at admission. Patients exhibiting elevated risk values for both thrombotic and inflammatory biomarkers received a score of 2, whereas those with high-risk values for only one or neither of these parameters were assigned scores of 1 and 0, respectively.

### 2.6 Statistical analysis

The enrolled patients were categorized into two groups based on their mRS scores: mRS < 3 and mRS ≥ 3 (Isaksson et al., 2020). Normally distributed continuous variables were represented by means ± SDs, while non-normally distributed continuous variables were represented by medians with interquartile ranges. Categorical variables were expressed as frequencies and percentages.

The evaluation of TIPS involved receiver operating characteristic (ROC) analysis, and the optimal cut-off values were determined using Youden’s index. Spearman’s correlation analysis was used to investigate the relationship between TIPS and the relative index of IS in patients with stroke. Between-group comparisons of categorical data were performed using the Chi-squared (χ^2^) test or Fisher’s exact test. Logistic regression analysis was performed to analyze the relationship between malnutrition and SAI. The logistic regression model was adjusted for risk factors which included sex, age, PSI, NIHSS, erythrocyte count, hemoglobin, leukocyte, platelet, albumin, creatinine, and triglyceride levels. The area under the ROC curve was established to evaluate the predictive ability of TIPS for death and unfavorable functional outcome in patients with stroke. In a subgroup analysis evaluating the effects of sex, age, alcohol consumption, smoking, hypertension, diabetes, WBC count, PLT, creatinine, PSI, A^2^DS^2^, and NRS2002, TIPS remained an independent predictor of unfavorable functional outcomes.

For all analyses, a two-tailed *P*-value < 0.05 was considered statistically significant. Statistical analyses were conducted using SPSS Statistics (version 25.0; SPSS, Chicago, IL, USA) and R Studio (version 4.1.3; Vienna, Austria).

## 3 Results

### 3.1 Baseline characteristics

A total of 915 patients met the inclusion criteria, with a mean age of 66 ± 13 years and 62.9% of the study sample being men. The three groups were defined as TIPS = 0 (*n* = 335), TIPS = 1 (*n* = 348), and TIPS = 2 (*n* = 232). Significant differences were observed in the demographic and clinical characteristics between the three groups.

Inflammatory markers (WBC count, neutrophil count, lymphocyte count, and NLR) and thrombus markers (PLT, D-dimer, INR, APTT, and fibrinogen) differed significantly between the three groups (*P* < 0.05). Additionally, the NIHSS, PSI, and A^2^DS^2^ demonstrated statistical differences between the three groups (*P* < 0.05) (Table 1).

**TABLE 1:** Baseline characteristics of patients with stroke.

	TIPS-0 (*n* = 335)	TIPS-1 (*n* = 348)	TIPS-2 (*n* = 232)	*P*-value
Female, *n* (%)	109 (32.5)	124 (35.6)	106 (45.7)	0.005
Age	61.0 (50.0–70.0)	68.0 (56.0–77.0)	70.0 (62.0–78.0)	<0.001
BMI	22.3 (6.90)	20.8 (8.57)	18.4 (10.6)	<0.001
Drinking, *n* (%)	122 (36.4)	91 (26.1)	63 (27.2)	0.007
Smoking, *n* (%)	167 (49.9)	136 (39.1)	77 (33.2)	<0.001
Hypertension, *n* (%)	210 (62.7)	193 (55.5)	139 (59.9)	0.153
Diabetes, *n* (%)	94 (28.1)	71 (20.4)	56 (24.1)	0.065
Etiological classification, *n* (%)				<0.001
Atherosclerosis	154 (46.0)	107 (30.7)	51 (22.0)	
Lacunar cerebral infarction	43 (12.8)	60 (17.2)	36 (15.5)	
Cardiogenic thrombus	25 (7.46)	23 (6.61)	22 (9.48)	
Other	77 (23.0)	64 (18.4)	32 (13.8)	
Unknown	36 (10.7)	94 (27.0)	91 (39.2)	
HGB, 10^9^/L	141 (132–152)	137 (124–148)	130 (117–143)	<0.001
PLT, 10^9^/L	185 (144–231)	172 (134–208)	171 (135–212)	0.015
WBC, 10^9^/L	6.30 (5.24–7.62)	7.18 (5.93–9.09)	8.95 (6.95–10.7)	<0.001
Albumin, g/L	43.1 (40.8–45.2)	42.0 (39.5–44.2)	40.9 (37.1–43.2)	<0.001
Lymphocyte, 10^9^/L	1.75 (1.44–2.20)	1.39 (1.01–1.86)	0.93 (0.76–1.19)	<0.001
Neutrophil, 10^9^/L	3.92 (3.17–4.92)	5.03 (3.79–7.01)	7.57 (5.64–9.07)	<0.001
NLR	2.27 (1.66–3.07)	3.54 (2.40–6.14)	7.30 (5.30–10.6)	<0.001
D-dimer, mg/L	0.31 (0.20–0.44)	0.98 (0.46–2.13)	2.17 (1.19–5.08)	<0.001
SII	404 (292–549)	613 (380–1030)	1307 (916–1847)	<0.001
APTT	27.1 (25.3–28.9)	26.3 (24.7–28.3)	26.1 (24.3–28.5)	0.001
INR	0.98 (0.93–1.05)	1.00 (0.94–1.08)	1.03 (0.96–1.09)	<0.001
Creatinine, μmol/L	72.0 (63.0–83.0)	75.0 (64.0–89.0)	71.0 (61.8–93.0)	0.131
LDL, mmol/L	2.37 (1.87–3.04)	2.44 (1.90–3.04)	2.36 (1.87–3.00)	0.725
HDL, mmol/L	1.15 (0.96–1.43)	1.21 (1.01–1.47)	1.22 (0.95–1.51)	0.232
Triglyceride, mmol/L	1.41 (0.98–2.02)	1.26 (0.93–1.84)	1.08 (0.86–1.56)	<0.001
Cys-C, mg/L	0.90 (0.82–1.03)	0.94 (0.81–1.12)	0.96 (0.81–1.14)	0.023
Fibrinogen, g/L	2.73 (2.34–3.20)	2.88 (2.39–3.49)	3.14 (2.48–4.11)	<0.001
Hospitalization, days	8.73 ± 4.99	10.7 ± 14.6	12.1 ± 12.3	0.002
NIHSS	4.00 (2.00–9.00)	6.00 (3.00–13.0)	12.0 (6.00–17.0)	<0.001
PSI	62.0 (50.0–72.0)	74.0 (60.8–89.0)	81.0 (67.0–99.0)	<0.001
A^2^DS^2^	4.00 (1.00–4.00)	4.00 (2.00–5.00)	5.00 (4.00–6.00)	<0.001
NRS2002	2.29 ± 1.20	2.49 ± 1.11	2.59 ± 1.30	0.010

BMI, body mass index; HGB, hemoglobin; PLT, platelet; WBC, white blood cell; NLR, neutrophil to lymphocyte ratio; SII, systemic immune-inflammation index; APTT, activated partial thromboplastin time; INR, international normalized ratio; LDL, low density lipoprotein; HDL, high density lipoprotein; Cys-C, cystatin C; NIHSS, National Institute of Health Stroke Scale; PSI, Pneumonia Severity Index; A^2^DS^2^, Age, Blood Pressure, Clinical Features, Duration of Symptoms, Diabetes, and Prior Stroke/TIA score; NRS2002, nutrition risk screening; mRS, modified Rankin Scale.

### 3.2 TIPS for death and unfavorable functional outcome

The predictive value of TIPS for death and unfavorable functional outcomes in patients with stroke is detailed in Table 2. The incidence of death and unfavorable functional outcomes increased proportionally with increasing TIPS doses. The predictive accuracy of unfavorable functional outcomes was 1.8- to 2.4-fold higher for patients with a TIPS of 1 or 2 than for those with a TIPS of 0. Similarly, the predictive accuracy of death was 3.6- to 6.0-fold higher for patients with a TIPS of 1 or 2 than for those with a TIPS of 0.

**TABLE 2:** The predictive value of thrombo-inflammatory prognostic score (TIPS) for death and unfavorable functional outcome in patients with stroke.

	Sensitivity, %	Specificity, %	Accuracy, %	PPV, %	NPV, %
**Unfavorable functional outcome**					
TIPS ≥ 0	100.00	0.00	29.29	29.29	–
TIPS ≥ 1	76.87	42.19	52.35	35.52	81.49
TIPS ≥ 2	43.28	82.07	70.71	50.00	77.75

TIPS, thrombo-inflammatory prognostic score; PPV, positive predictive value; NPV, negative predictive value.

Univariate logistic regression models, as presented in Table 3, demonstrated an association between TIPS and death, as well as unfavorable functional outcomes in patients with stroke. Furthermore, an increase in TIPS remained independently associated with death and unfavorable functional outcomes, even after adjusting for latent variables in the multivariate logistic regression analysis. For unfavorable functional outcome, the odds ratios (OR) of 1.060 (95% CI: 0.702–1.599, *P* = 0.782) for TIPS 1 vs. 0 and 2.480 (95% CI: 1.503–4.091, *P* < 0.001) for TIPS 2 vs. 0 and death, the ORs were 1.741 (95% CI: 0.914–3.316, *P* = 0.092) for TIPS 1 vs. 0 and 3.429 (95% CI: 1.650–7.126, *P* = 0.001) for TIPS 2 vs. 0 (Table 3).

**TABLE 3:** Logistic regression analysis regarding correlations between TIPS and clinical outcomes.

Variables	TIPS 1 vs. 0 OR (95% CI)	*P*-value	TIPS 2 vs. 0 OR (95% CI)	*P*-value
**Unfavorable functional outcome**				
Unadjusted	1.536 (1.066–2.214)	0.021	4.403 (3.02–6.421)	0
Adjusted*	1.06 (0.702–1.599)	0.782	2.48 (1.503–4.091)	0
**Death**				
Unadjusted	2.429 (1.348–4.377)	0.003	6.093 (3.438–10.797)	0
Adjusted*	1.741 (0.914–3.316)	0.092	3.429 (1.65–7.126)	0.001

*Risk factors adjustment included gender, age, PSI, Pneumonia Severity Index, NIHSS, erythrocyte count, hemoglobin, leukocyte, platelet, albumin, creatinine, and triglyceride. TIPS, thrombo-inflammatory prognostic score; NIHSS, National Institute of Health Stroke Scale; OR, odds ratio; IC, confidence interval.

### 3.3 The predictive capacity of TIPS for various outcomes in stroke patients

**FIGURE 1:**
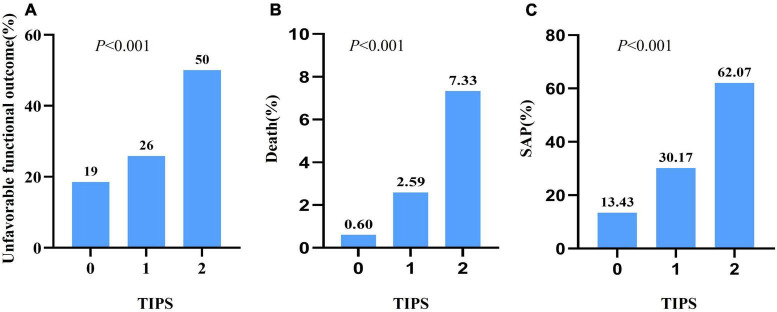
The unfavorable functional outcome **(A)**, death **(B)**, and SAP **(C)** with different TIPS scores in patients with stroke.

**FIGURE 2:**
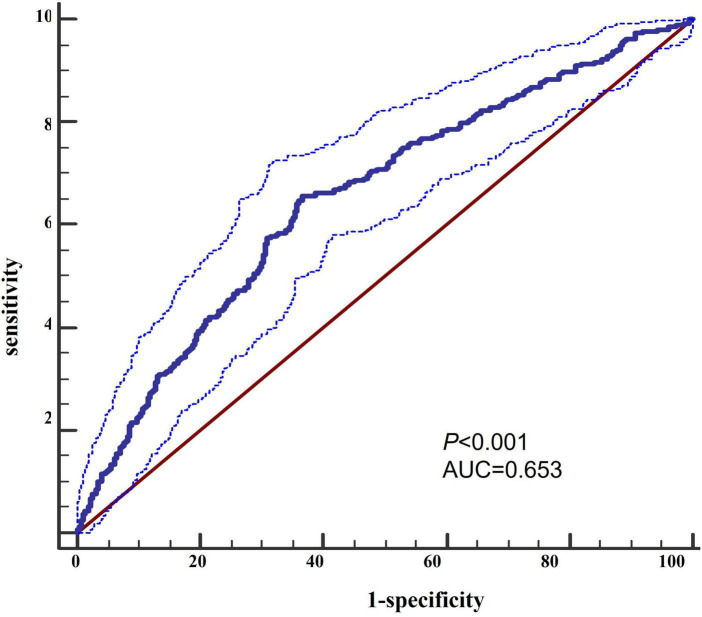
Receiver operating characteristic curve for TIPS of the unfavorable functional outcome.

As depicted in Figure 1, a TIPS score of 2 was associated with a twofold increase in unfavorable functional outcomes compared with a TIPS score of 0. Moreover, the risk of death exhibited a substantial increase, with a TIPS of 2 showing a 12-fold increase in comparison with a TIPS of 0. Additionally, a TIPS score of 2 is 4.6 times more likely to be linked with SAP than a TIPS score of 0. The ROC curve for TIPS regarding unfavorable functional outcomes revealed an area under the curve of 0.653 (Figure 2).

### 3.4 Comparison between TIPS and the relative index of IS

**FIGURE 3:**
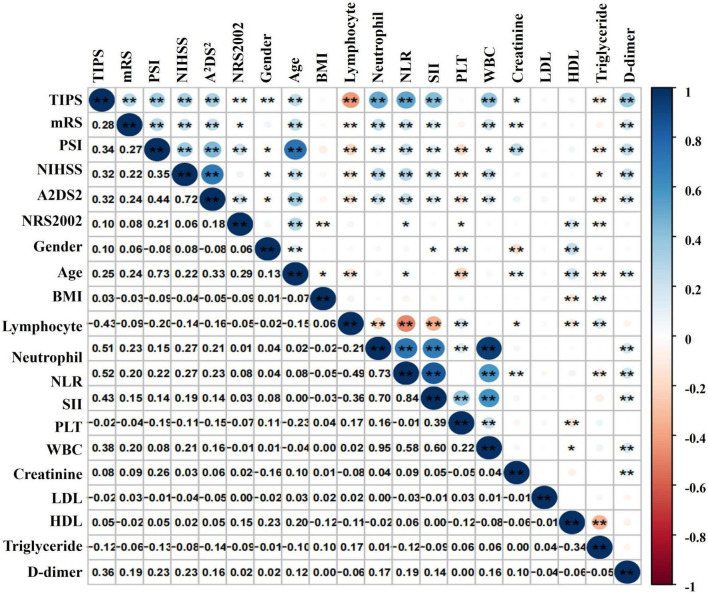
Correlation analysis of TIPS and the relative index of IS in patients with stroke. *P < 0.05, **P < 0.01.

Pearson correlation analysis revealed associations between TIPS and various factors, including NIHSS score, PSI, A^2^DS^2^, neutrophil count, NLR, SII, WBC count, D-dimer, and lymphocyte count. Notably, TIPS exhibited the strongest correlations with lymphocyte count and NLR (Figure 3).

### 3.5 Subgroup analysis

In subgroup analysis to evaluate the effects of sex, age, alcohol consumption, smoking, hypertension, diabetes, WBC count, PLT, creatinine, PSI, A^2^DS^2^, and NRS2002, TIPS remained an independent predictor of unfavorable functional outcomes (Table 4).

**TABLE 4:** Subgroup analysis of the association between TIPS and unfavorable functional outcome by multivariate logistic regression analysis.

Variables	TIPS 1 vs. 0 OR (95% CI)	*P*-value	TIPS 2 vs. 0 OR (95% CI)	*P*-value	*P* for interaction
Gender					0.175
Male	1.309 (0.789–2.169)	0.297	2.567 (1.329–4.957)	0.005	
Female	0.696 (0.33–1.466)	0.340	2.329 (1–5.42)	0.050	
Age					0.194
<60	1.34 (0.56–3.206)	0.511	3.949 (1.191–13.092)	0.025	
≥60	0.908 (0.565–1.461)	0.691	2.155 (1.224–3.793)	0.008	
Drinking					0.928
No	0.951 (0.574–1.575)	0.845	1.884 (1.018–3.486)	0.044	
Yes	1.326 (0.625–2.814)	0.462	5.101 (1.868–13.93)	0.001	
Smoking					0.698
No	0.819 (0.465–1.443)	0.490	1.966 (1.007–3.835)	0.048	
Yes	1.412 (0.755–2.64)	0.280	3.62 (1.576–8.314)	0.002	
Hypertension					0.416
No	1.38 (0.686–2.776)	0.367	3.253 (1.411–7.496)	0.006	
Yes	0.908 (0.533–1.546)	0.721	2.701 (1.377–5.297)	0.004	
Diabetes					0.426
No	0.908 (0.533–1.546)	0.721	2.701 (1.377–5.297)	0.004	
Yes	0.525 (0.208–1.324)	0.172	1.533 (0.478–4.92)	0.472	
WBC, 10^9^/L					0.120
≤10	0.837 (0.537–1.304)	0.431	2.301 (1.377–3.845)	0.001	
>10	2.518 (0.653–9.719)	0.180	3.321 (0.811–13.597)	0.095	
PLT, 10^9^/L					0.654
≤100	7.581 (0.945–60.795)	0.057	5.039 (0.481–52.789)	0.177	
>100	0.944 (0.622–1.431)	0.785	2.107 (1.317–3.373)	0.002	
Creatinine, μmol/L					0.250
≤115	0.98 (0.647–1.485)	0.924	2.385 (1.489–3.82)	<0.001	
>115	2.272 (0.167–30.937)	0.538	0.327 (0.012–8.951)	0.508	
PSI					0.033
≤90	0.954 (0.608–1.496)	0.836	2.37 (1.387–4.051)	0.002	
>90	1.196 (0.42–3.409)	0.737	2.028 (0.693–5.931)	0.197	
A^2^DS^2^					0.461
≤4	0.821 (0.486–1.386)	0.460	2.384 (1.258–4.519)	0.008	
>4	1.223 (0.604–2.478)	0.576	2.574 (1.224–5.414)	0.013	
NRS2002					0.893
≤2	1.047 (0.57–1.925)	0.883	2.507 (1.234–5.092)	0.011	
>2	0.922 (0.529–1.61)	0.776	1.775 (0.947–3.326)	0.073	

TIPS, thrombo-inflammatory prognostic score; WBC, white blood cell; PLT, platelet; PSI, Pneumonia Severity Index; A^2^DS^2^, Age, Blood Pressure, Clinical Features, Duration of Symptoms, Diabetes, and Prior Stroke/TIA score; NRS2002, nutrition risk screening; OR, odds ratio; IC, confidence interval.

### 3.6 Mediation analysis

In the mediation analysis, TIPS exhibited a more substantial effect as a mediator between the NIHSS score and clinical outcomes, with an effect ratio of 37.5%. This effect was greater than that of the D-dimer level (effect ratio, 12.5%) and NLR (effect ratio, 25%) (Table 5).

**TABLE 5:** Direct and indirect effects of D-dimer, NLR, and TIPS on NIHSS and clinical outcomes.

Variables	Path	Effect	Effect ratio (%)	Boot 95% CI	*P*	SE	*Z*
**Unfavorable functional outcome**							
D-dimer	Indirect	0.001	12.50	(0.001–0.002)	0.053	0	1.939
NIHSS	Direct	0.007	87.50	(0.004–0.009)	<0.001	0.001	4.487
	Total	0.008	100.00	(0.005–0.010)	<0.001	0.001	5.312
**Inflammation**							
NLR	Indirect	0.002	25.00	(0.001–0.003)	0.005	0.001	2.808
NIHSS	Direct	0.006	75.00	(0.003–0.009)	<0.001	0.002	3.884
	Total	0.008	100.00	(0.005–0.010)	<0.001	0.001	5.336
**Thrombus combined inflammation**							
TIPS	Indirect	0.003	37.50	(0.002–0.004)	<0.001	0.001	4.489
NIHSS	Direct	0.005	62.50	(0.002–0.008)	0.001	0.002	3.176
	Total	0.008	100.00	(0.005–0.010)	<0.001	0.001	5.428

β Coefficient was calculated by standard regression equation. TIPS, thrombo-inflammatory prognostic score; NLR, neutrophil to lymphocyte ratio; IC, confidence interval; NIHSS, National Institute of Health Stroke Scale.

### 3.7 Survival analysis with different TIPS

**FIGURE 4:**
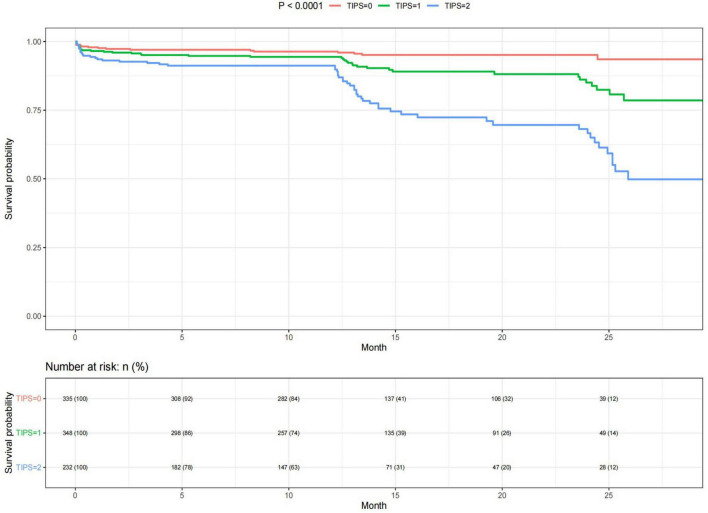
Kaplan–Meier curves of different TIPS in patients with stroke.

The Kaplan–Meier curves illustrated a decrease in the survival probability of patients with increasing TIPS. Furthermore, the survival probability of patients with a TIPS score of 1 was observed to be twice as high as that of patients with a TIPS score of 2 (Figure 4).

## 4 Discussion

Our study highlighted a significant association between elevated TIPS, derived from thrombo-inflammatory biomarkers, and increased risks of death and unfavorable functional outcomes in patients with IS. Notably, elevated TIPS emerged as an independent predictor of these adverse outcomes, even after adjusting for confounding factors. We observed that TIPS had a more substantial impact on the NIHSS score and clinical outcomes compared to that by individual biomarkers like D-dimer and NLR. As TIPS scores escalated, there was a notable rise in the proportion of patients experiencing unfavorable functional outcomes, death, and SAP, underlining its value as a prognostic indicator in IS.

The primary causes of death and disability in patients with stroke are prolonged interruptions in the cerebral blood supply, delayed or insufficient medical interventions, and post-stroke complications (Feske, 2021). Early assessment becomes crucial for timely clinical intervention, and the inflammatory response plays a pivotal role in the onset and progression of stroke (Miller and Behrouz, 2016; DeLong et al., 2022). Various inflammatory markers, including neutrophil levels and NLR, have demonstrated a strong predictive value for IS prognosis (Sharma et al., 2021; Tirandi et al., 2023). Notably, these findings emphasize the significance of understanding and monitoring inflammatory markers for effective stroke prognosis and management (Bui et al., 2022; Denorme et al., 2022). Thrombosis plays a significant role in influencing the occurrence and prognosis of stroke. Biomarkers such as D-dimer and INR are crucial contributors to our understanding of IS (Liu et al., 2020; Ohara et al., 2020). The intricate interplay between thrombosis and inflammation at both the cellular and molecular levels establishes a thrombotic-inflammatory state that is closely associated with the severity and complications of IS (Nagareddy and Smyth, 2013). The assessment of prognosis in patients with IS solely based on a single thrombus or inflammatory marker is challenging. Therefore, adopting a strategy that involves multiple biomarkers linking the inflammatory status to thrombotic markers could provide additional predictive insights into the risk of unfavorable functional outcomes. This approach may surpass the utility of relying solely on a single inflammatory or thrombotic biomarker to understand the complex dynamics of IS prognosis. Previous studies have demonstrated the efficacy of combining TIPS biomarkers to stratify the risk of adverse clinical outcomes in sepsis patients (Li et al., 2020). Notably, the implementation of TIPS based on D-dimer and PCT levels has improved the risk stratification of patients with sepsis (Li et al., 2018). Moreover, TIPS has proven to be a valuable tool for the early identification of high-risk patients for SAP after IS (Li et al., 2023).

The utility of TIPS extends to predicting adverse clinical outcomes in various conditions, including patients with acute pancreatitis and those with type B acute aortic dissection, enabling a 28-day prognosis (Li et al., 2017; Han et al., 2022). Our study revealed that TIPS has an enhanced predictive value for IS. These findings underscore the versatility and potential applicability of TIPS in diverse clinical scenarios to improve prognostic assessment.

While individual biomarkers provide valuable information, relying solely on one marker for prognosis can be challenging due to the complexity of IS dynamics. Our study introduced TIPS, a composite score combining inflammatory and thrombotic markers, which offers enhanced prognostic insights compared to single markers. Individuals with stroke exhibit an increased burden of predisposing risk factors that can worsen their prognosis, leading to heightened systemic inflammation and thrombotic tendencies. This interplay emphasizes the complexity of factors influencing stroke outcomes and underscores the importance of considering multiple markers for a more comprehensive prognostic evaluation.

Existing evaluation tools rely primarily on assessing the degree of neurological damage and stroke risk factors (Joundi and Menon, 2021; Ekker et al., 2023). However, because risk factors only capture a patient’s clinical background, conducting dynamic evaluations may be challenging, leading to difficulties in dynamically assessing patient prognosis. In contrast, this study was based on a comprehensive evaluation of the pathological and physiological processes involving inflammation and thrombosis in stroke injuries. By doing so, it aims to predict patient prognosis more effectively and facilitate a dynamic assessment of a patient’s overall outlook.

The study has several limitations that need to be acknowledged. First, this was a retrospective cohort study conducted at a single center, which restricted our ability to establish a causal relationship between TIPS and its associated thrombo-inflammatory biomarkers. Second, we did not assess the predictive utility of TIPS within subgroups of patients with IS, considering factors such as varying treatment modalities, time intervals between onset and hospitalization exceeding 6 h, and other specific subpopulations. Third, other inflammatory metrics were not included in the TIPS score, potentially limiting the comprehensive evaluation of inflammatory processes. Finally, this study did not investigate the time required for the completion during hospitalization in the emergency department. Therefore, further validation through multicenter prospective studies is essential to validate the predictive capacity of TIPS for IS, as well as to assess its practical applicability across diverse clinical settings.

## 5 Conclusion

Our study offers insights into the predictive value of TIPS for IS based on thrombo-inflammatory biomarkers. These findings suggest that as TIPS increases, the incidence of adverse outcomes escalates in parallel. This underscores the potential utility of TIPS as a prognostic tool for assessing and predicting adverse clinical outcomes in patients with IS.

## Data availability statement

The original contributions presented in this study are included in the article/supplementary material, further inquiries can be directed to the corresponding authors.

## Ethics statement

Ethical approval was not required for the study involving humans in accordance with the local legislation and institutional requirements. Written informed consent to participate in this study was not required from the participants or the participants’ legal guardians/next of kin in accordance with the national legislation and the institutional requirements.

## Author contributions

XZ: Methodology, Writing – original draft, Writing – review & editing. LL: Data curation, Visualization, Writing – original draft. YL: Formal analysis, Writing – original draft. NH: Software, Writing – original draft. JW: Data curation, Writing – original draft. YG: Supervision, Writing – review & editing. HL: Funding acquisition, Project administration, Writing – review & editing. DL: Conceptualization, Funding acquisition, Methodology, Project administration, Validation, Writing – review & editing.
